# Real-world data to improve organ and tissue donation policies: lessons learned from the tissue and organ donor epidemiology study

**DOI:** 10.1186/s12961-024-01237-0

**Published:** 2024-11-12

**Authors:** Melissa A. Greenwald, Hussein Ezzeldin, Emily A. Blumberg, Barbee I. Whitaker, Richard A. Forshee

**Affiliations:** 1 Uniformed Services University, Bethesda, MD USA; 2 Present Address: MA Greenwald Consulting, Chicago, IL USA; 3 Center for Biologics Evaluation and Research, United States Food and Drug Administration, Silver Spring, MD USA; 4 Division of Infectious Diseases, Department of Medicine, Perelman School of Medicine, University of Pennsylvania, Philadelphia, PA USA; 5 American Association of Tissue Banks, McLean, VA, USA

**Keywords:** Organ transplantation, Tissue transplantation, Ocular transplantation, Communicable diseases, Donor screening, Donor testing, Real world data, Health information exchange, Interoperability, Patient safety, Traceability

## Abstract

**Background:**

The transplantation of human organs, and some human tissues, is often the only life-saving therapy available for serious and life-threatening congenital, inherited or acquired diseases. However, it is associated with a risk of transmission of communicable diseases from donor to recipient. It is imperative to understand the characteristics of the donor population (including both potential and actual donors) to inform policies that protect recipient safety. The Tissue and Organ Donor Epidemiology Study (TODES) was a pilot project designed to identify and collect standardized information on deceased persons referred for organ, tissue and/or eye donation, and to estimate (to the extent possible) infectious disease prevalence and incidence of human immunodeficiency virus (HIV), hepatitis B virus (HBV) and hepatitis C virus (HCV) in this population. TODES is summarized here to shed light on addressable limitations on accessing data needed for transplant recipient safety. Limitations, future research needs and potential pathways to solve the remaining data needs are explored.

**Methods:**

Retrospective data for all deceased donors during a 5-year period from 2009 to 2013 were obtained from participating organ procurement organizations (OPOs), tissue establishments and eye banks. These decedent data were used to ascertain whether the available real-world data (RWD) could be used to inform donor screening and testing policy.

**Results:**

The TODES database contains 291 848 records received from nine OPOs and 42 451 records received from four eye banks. Data were analysed from deceased donors with at least one organ, tissue or ocular tissue recovered with the intent to transplant. Results for potential donors were not analysed. Available RWD at the time of the TODES study were not fit-for-purpose to help characterize the organ, tissue and eye donor populations and/or to inform donor screening policy.

**Conclusions:**

Recent advances in electronic data collection systems make it more realistic to now collect fit-for-purpose RWD that address the research needed to improve transplant safety.

**Supplementary Information:**

The online version contains supplementary material available at 10.1186/s12961-024-01237-0.

## Background

Human organ, tissue and eye (OTE) transplants provide tremendous individual and societal benefits. However, they are also associated with the risk of communicable disease transmission to OTE transplant recipients. Many risk mitigation strategies are currently in place, including extensive donor screening and testing for active and latent infections. Nevertheless, donor-derived infections have occurred [1–8] and are not anticipated to be fully preventable, highlighting the need for continued reassessment of how to best evaluate donors to optimally balance safety with availability. Determining what communicable disease hazards pose the most risk to recipients, and therefore merits donor screening or testing measures, requires understanding what hazards exist on the front end of the donation process (that is, epidemiology of communicable diseases in the donor population), in addition to understanding of the communicable disease transmissions that occur despite donor evaluation and tissue processing. Data regarding the background rates of communicable diseases in OTE donor populations are unavailable, and in the USA, tissue and eye transmission events are informed solely by a passive surveillance system. Without the ability to measure the rates of positive communicable disease test results present in the donor population to determine the expected rates of infection within OTE donor populations, it is not possible to follow trends over time and respond to changes in the epidemiology of potential donors, or identify problems with donor assessment tools (for example, donor screening assays and donor history questionnaires). The need for such data as part of a biovigilance system has been emphasized by federal and non-federal stakeholders alike in public workshops and white papers (Table 1).

**Table 1:** Summary of forums by federal and non-federal stakeholders and recommendation and findings

Forum	Recommendation
The joint CDC, FDA and HRSA 2005 workshop [32]	Communication networks should be improved A unique donor identifier for both organs and tissues should be created Education and dissemination of information to clinicians and transplant patients should be strengthened A framework for clinicians to report transplant-associated adverse events should be clearly delineated A notification algorithm for tracking among and between organs and tissues should be designed
Advisory Committee on Blood Safety and Availability (ACBSA) 2006 [33, 34]	Called for a public–private partnership “biovigilance” initiative to collect analyse and report on the outcomes of collection and transfusion and/or transplantation of blood components and derivatives, cells, tissues and organs
2007 workshop, “Organ and Tissue Safety Workshop 2007: Advances and Challenges” [35]	Review the epidemiology of transmission of infection and malignancy associated with allografts (that is, organs, tissues and eyes) Understand existing reporting standards and requirements, and enhance communication regarding safety issues between the organ and tissue communities, regulatory agencies and other stakeholders Evaluate progress on development of the TTSN, and other interventions to detect and prevent transmission events Examine advances in diagnostic and screening technologies that could be applied to the enhancement of safety of allograft transplantation
2009 PHS White Paper [33]	Identified 8 gaps for biovigilance in HCT/P and solid organ. Given the policy challenges, the recommendations were: Government resource support for a national biovigilance program to monitor and enhance safety of blood, organs and HCT/Ps Integration of systems within the government and those within the private sector, involving blood, organs and HCT/Ps, including all related voluntary and mandatory adverse event reporting systems Enhance mechanisms for surveillance, including sentinel reporting and investigation, and comprehensive surveillance that features benchmarking
2010 Emerging Infectious Diseases Workshop [5, 36]	The unknown sensitivity and specificity of current medical and behavioural history tools to screen donors for risk factors associated with infectious agents The difficulty in distinguishing acute from chronic or persistent infections using standard testing modalities, especially given the prolonged window period of many serological assays and the limited sensitivity and specificity of NAT for some infections, especially those acquired within days of donation The limited ability of NAT to identify infections not associated with active bloodstream involvement The variability in performance between different assays, including those used for donor screening and those used for diagnostic reasons, where performance characteristics have not been evaluated in the deceased donor setting; this may limit the ability of transplant personnel to compare and interpret some tests
2013 PHS guideline to improve organ recipient outcomes [37]	Updated 1994 guideline that covered only HIV in organs and tissues Reduce the risk of HIV, HBV and HCV transmission through organ transplantation Gaps in the literature and quality of evidence affected the ability to reach firm conclusions for organs in certain interventions Emphasized the need for putting a system in place allowing tracking between a common deceased donor and (1) recovered organs, (2) recovered associated blood vessel conduits and (3) recovered tissues and eyes to facilitate notification when a donor-derived disease transmission is suspected Further research was recommended in numerous areas, including estimating incidence and prevalence of HIV, HBV and HCV among deceased donors, and developing standardized algorithms for discrimination of initially reactive (positive) organ donor immunoassay and NAT results
2020 PHS Guideline Assessing Solid Organ Donors and Monitoring Transplant Recipients for Human Immunodeficiency Virus, Hepatitis B Virus and Hepatitis C Virus Infection [18, 38–40]	Updated evidence review of recent organ-transplant–specific evidence, intended to increase the use of organs while continuing to maintain transplant recipient safety Changes from the 2013 PHS guideline: Identifying a timeframe for recipient pretransplant testing Updating the criteria for identifying donors at risk for undetected donor HIV, HBV or HCV infection; the removal of any specific term to characterize donors with HIV, HBV or HCV infection risk factors; universal organ donor HIV, HBV and HCV nucleic acid testing; and universal posttransplant monitoring of transplant recipients for HIV, HBV and HCV infections Removing the following risk criteria, as it is no longer applicable for assessing potential disease transmission from donors to recipients: Woman who has had sex with a man who has had sex with another man Newly diagnosed or treated syphilis, gonorrhoea, chlamydia or genital ulcers Hemodialysis Hemodiluted blood specimen used for donor HIV, HBV and HCV testing Child (aged ≤ 18 months) born to a mother at increased risk for HIV, HBV or HCV infection Child breastfed by a mother at increased risk for HIV infection

*TTSN* Transplantation Transmission Sentinel Network, *CDC* Centers for Disease Control and Prevention, *HCT/P* Human cells tissues and cellular and tissue-based products, *NAT* nucleic acid test, *HBV* hepatitis B virus, *HIV* human immunodeficiency virus, *HCV* hepatitis C virus, *HRSA* Health Resources and Services Administration, *PHS* Public Health Services, *FDA* Food and Drug Administration

The Department of Health and Human Services (HHS) is charged with taking measures to minimize the risk of transmission of disease from donated OTE while optimizing product availability through relevant agencies, including the Health Resources and Services Administration (HRSA), Centers for Disease Control and Prevention (CDC) [9], Food and Drug Administration (FDA),^1^ and Centers for Medicare and Medicaid Services (CMS). From 2012 to 2016, the Department of Health and Human Services (HHS) conducted the Tissue and Organ Donor Epidemiology Study (TODES) [10], a demonstration project designed to evaluate the ability to identify and collect data on deceased persons referred for OTE donation (that is, potential donors) in a standardized manner. TODES explored using existing data sources [namely, real-world data (RWD)] [11] to determine whether these databases were suitable, or “fit-for-purpose”, to provide data for assessing the epidemiology of currently acknowledged communicable disease risk factors among donors. TODES assessed the ability to collect data on potential donors by determining whether it would be possible to estimate the prevalence and (to the extent possible) incidence of human immunodeficiency virus (HIV), hepatitis B virus (HBV) and hepatitis C virus (HCV). Without baseline epidemiological data of OTE donors, including the rate of expected positive test results, it is not possible to identify changes in those baseline rates that may be an indicator of such problems as assay performance, shifting epidemiology or usefulness of current testing algorithms. If the available data sources were not fit-for-purpose, TODES would identify those gaps, informing regarding future needs to address those gaps.

TODES provides a broad overview of the OTE transplantation data collection infrastructure, highlights the complexity of the OTE donation processes, identifies gaps in data collection systems and underscores the challenges to implementing standardized donor data collection or the ability to perform post-hoc analysis. Bridging these gaps is important, especially given that donors may donate OTE within systems that are unable to identify and communicate transplantation transmission risks effectively and efficiently. While TODES specifically evaluated the ability to collect positive and negative test results among potential OTE donors, the study’s limitation in achieving this underscores critical gaps in the OTE transplantation data infrastructure, particularly in its ability to support effective biovigilance. For example, recent *Mycobacterium tuberculosis* (MTB) transmissions via human bone tissue containing viable cells [2, 8] demonstrated that the current donor screening practices failed to prevent TB transmission. Unfortunately, there is no clear pathway to assess the frequency of MTB or its risk factors in the current system, nor is it clear how such information might impact proposed or actual policy changes within OTE donor populations. There were significant challenges faced in identifying all recipients of the contaminated product [12], which further resulted in difficulties identifying secondary transmissions to healthcare workers [13], highlighting the need for improved data collection capacity within the entire donation and transplantation process.

In this manuscript, we provide an overview of the transplant process, explain the TODES approach in collecting RWD and highlight relevant findings from the collected data. Given the lessons learned from TODES, we explore current strategies and practical solutions that can pave the way for future studies and improve the balance between safety and availability of OTE transplantation.

## Methods

### Study overview

The HHS-sponsored TODES study had three overarching goals: (1) develop a study design or framework to effectively collect and analyse demographic, screening and infectious disease testing data obtained from deceased OTE donors, including referral-only donors, in a standardized manner; (2) identify challenges to obtaining such data in a consistent and standardized format; and (3) identify limitations and sources of biases from data captured in this study. HHS funding was announced in 2011, the study contract was awarded in 2012, and the final report was published in July 2016 [10]. The TODES working group (WG) comprised multiple federal and non-federal stakeholders with experience in OTE transplantation. Prospective OTE establishments were contacted for recruitment. Characteristics of potential study participants are listed in Table 2.

**Table 2:** Characteristics of TODES participant records by organization type

Organization type	Organization code	All records	Referrals only records	Records representing potential donors *n*	Records with UNOS data/OPO-linked data *n*	Record with any test results (*n*)	Organ donor records with any test results (*n*)	Tissue donor records with any test results (*n*)	Eye donor records with any test results (*n*)	Referrals – only records with any test results (*n*)
OPO	A	5611	1455	4156	751	1158	751	691	491	104
	B	169 760	164 347	5413	1381	1716	1384	663	2	238
	c	3967	42	3925	652	3949	652	3621	0	33
	D	1867	2	1865	618	1860	618	1370	562	2
	F	8302	0	8302	1135	8,299	1134	7636	4196	0
	I	4234	235	3999	1366	4060	1371	3180	345	117
	K	760	82	678	644	644	644	205	199	0
	L	96 386	89 703	6683	724	870	725	359	252	101
	M	961	61	900	870	954	870	380	318	60
	**Total**	**291 848**	**255 927**	**35 921**	**8141**	**23 510**	**8149**	**18105**	**6365**	**655**
Eye bank	E	23 044	0	23 044	0	22 924	0	9266	22 924	0
	G	11 083	417	10 666	0	9873	0	0	9663	210
	H	2644	70	2574	0	2500	0	0	2434	66
	J	5680	0	5680	0	5168	203	2399	5168	0
	**Total**	**42 451**	**487**	**41 964**	**0**	**40 465**	**203**	**11 665**	**40 189**	**276**

Source: adapted from TODES report

Referrals-only records; UNOS Matches records; records with any test results; and organ, tissue and eye records with any test results. Based on all records received; includes known duplicates that were removed in subsequent analyses. The organ, tissue and eye donors with any test results are not mutually exclusive, so the record totals may add up to a value greater than that reported in the records with any test results column. OPO B and Eye Bank E were the only participants that submitted records that could be linked. Linkage of the 169 760 records submitted by OPO B and the 23 044 records submitted by Eye Bank E, resulted in 7534 records in each dataset that were determined to be matching organ and/or tissue/eye donors. For organs, information about potential donors, also called “referrals-only records,” is entered into one of the OPO’s computer systems and is assigned a UNOS identifier (ID). However, if the donor is found ineligible to donate organs, the donor records are not shared with UNOS and therefore would be unavailable in the OPTN dataset; if eligible for tissue donation, a UNOS ID is used, and other donor ID coding is also used. The Referrals-only records column contains records with no indication of donor type (organ, tissue or eye), defined to have not resulted in donation

*OPO* organ procurement organization, *UNOS* United Network for Organ Sharing, *OPTN* Organ Procurement and Transplantation Network

A staff member from each participating establishment provided feedback about their organization, data collection methods and donor population. As noted in Table 3, some potential participants were unable to provide the data required or were unable to participate for other reasons. Significant variation in the practice of obtaining information about testing for infections impacted the ability to accrue accurate and reliable RWD. As a result of the provided feedback, several sources of retrospectively deidentified RWD were obtained and reviewed for both referred potential donors and actual donors during the 5-year period from 2009 to 2013.

**Table 3:** Prospective TODES participants by OPTN region and final participant types

OPTN region	Number and type of prospective participants	Number and type of final participants^a^
1	1 (one OPO)	9 OPOs 4 eye banks
2	2 (two OPOs)	
3	5 (four OPOs; one eye bank)	
4	2 (two OPOs)	
5	4 (three OPOs; one eye bank)	
6	2 (one OPO; one eye bank)	
7	2 (two OPOs)	
8	2 (one OPO; one eye bank)	
9	1 (one OPO)	
10	4 (two OPOs; two eye banks)	
11	4 (two OPOs; two eye banks)	

Initial information was obtained from 29 potential study participants: 21 OPOs selected by AOPO and work group participants representing all 11 OPTN regions, and eight eye banks identified by EBAA. While six of the largest tissue processors also discussed their process of donor data collection, it is notable the working group determined that due to lack of a common donor identification across organ, eye and tissues, obtaining data directly from tissue processors or from testing laboratories would likely result in duplicate donor data reporting. After RWD sources were identified for the study, 13 study participants were able to provide tissue donor-level data for analysis (9 OPOs and 4 eye banks)

^a^Final participants’ OPTN regions were not provided

*EBAA* Eye Bank Association of America, *OPO* organ procurement organization, *OPTN* Organ Procurement and Transplantation Network, *RWD* real-world data

The study also collected retrospective data from 2009 through 2013 for decedent donors. The final organ donor data were obtained from the Organ Procurement and Transplantation Network (OPTN) and organ procurement organizations (OPOs). While the final tissue donor data were obtained from eye banks and OPOs that recovered tissue, no usable data were available from tissue establishments (TEs) in that duplicate results were unable to be excluded when tissue recovered from a single donor is commonly sent to more than one tissue processor, each of whom uses different donor identification numbers from one another. These data included basic demographic information, limited medical history and behavioural risk data, and infectious disease test results, if available. A data dictionary was developed, data were obtained, a TODES database was established and data were analysed. The data dictionary and other details about the study are available for review in the original study report [10].

### Overview of organ, eye and tissue transplantation process

**Fig. 1:**
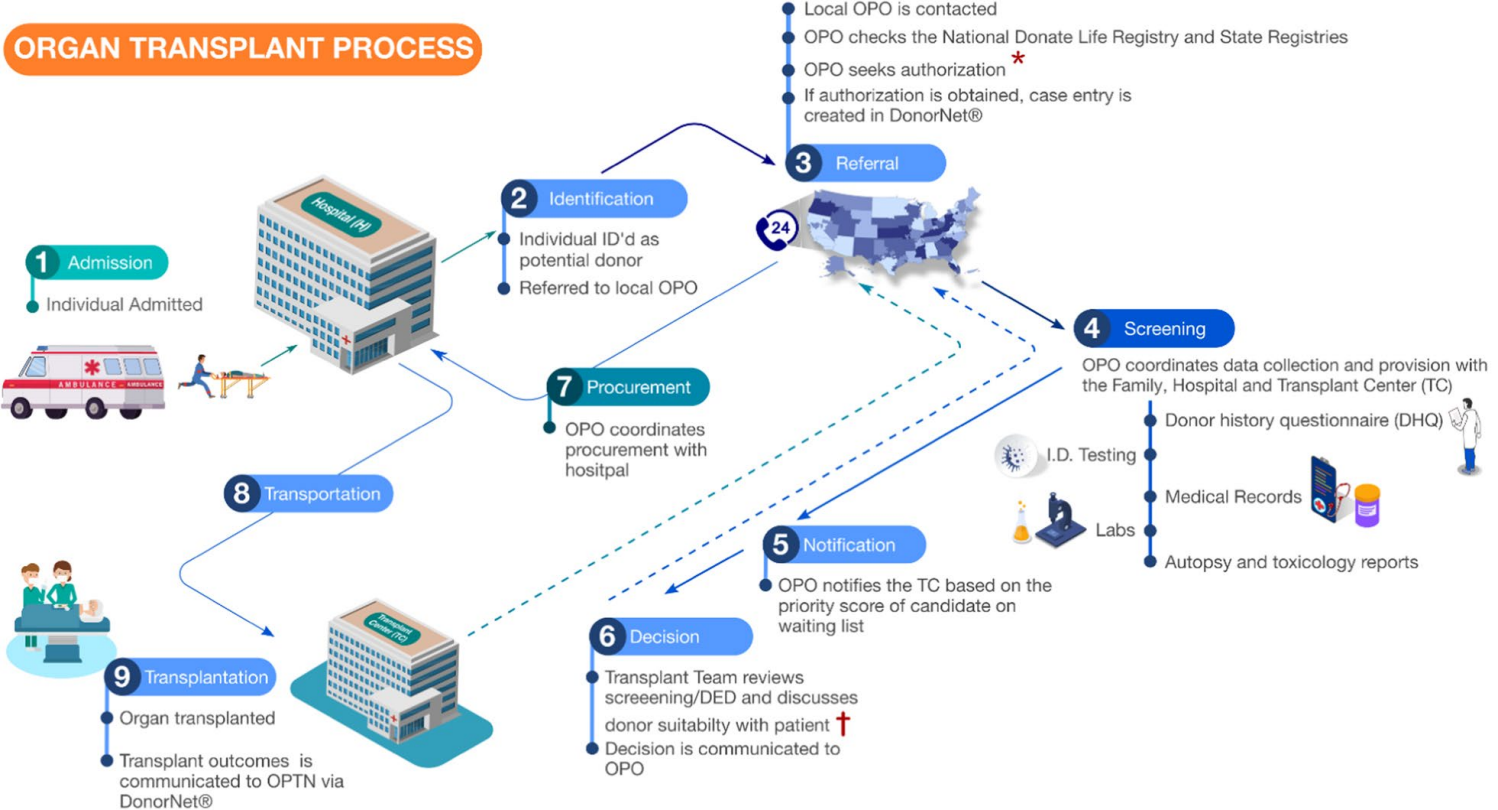
Steps involved in organ transplant. Organ transplant process simplified into nine steps includes: (1) admission, (2) identification, (3) referral, (4) screening, (5) notification, (6) decision, (7) procurement, (8) transportation and (9) transplantation. Solid lines denote forward data flow/communication. Dashed lines denote feedback to OPOs. ^*^OPO checks state and national donor registries as mandated by law to honour first-person authorization. ^†^Donor eligibility determination (DED), including donor screening and testing, where data about the donor risk are collected from family, medical records, infectious disease testing, etc. *OPO* Organ Procurement Organization, *OPTN* Organ Procurement and Transplantation Network, *TC* transplant centre, *DHQ* donor history questionnaire

**Fig. 2:**
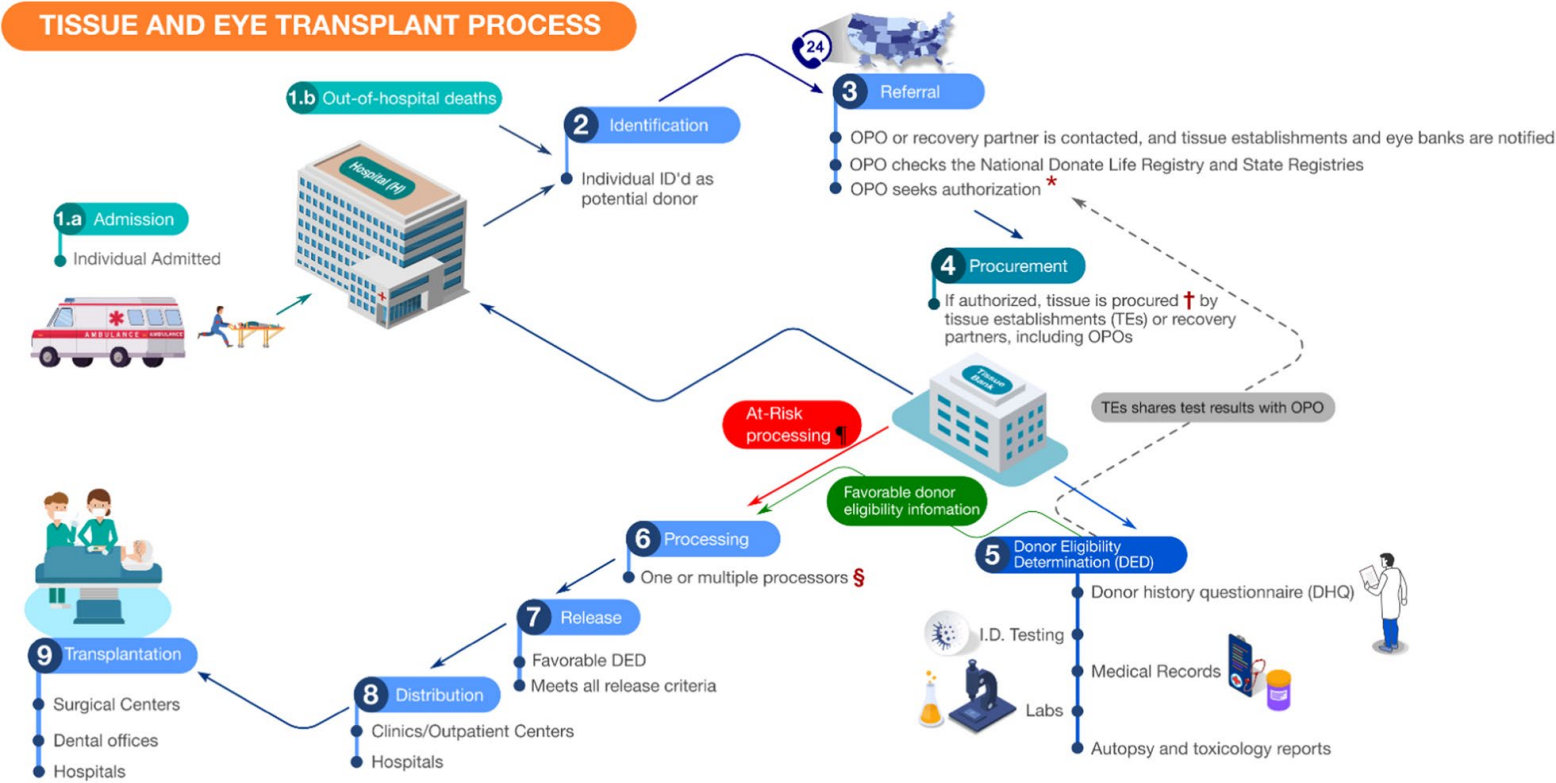
Steps involved in tissue and eye transplant. The tissue and eye transplant process simplified into nine steps includes: (1a) admission and (1b) out-of-hospital-deaths (at-home, or by medical examiner/coroner), (2) identification, (3) referral, (4) procurement, (5) donor eligibility determination (DED), (6) processing, (7) release, (8) distribution and (9) transplant. Solid lines denote forward data flow/communication. Dashed line denotes feedback to OPOs/ recovery partners. ^*^OPO/recovery partners check state and national donor registries as mandated by law to honour first-person authorization and notify families of the individual’s registration status; families are contacted for authorization in the absence of first-person donor registration. ^†^Tissue and eye procurement could take place in the hospital, funeral home, medical examiner/coroner office or OPO procurement centre. In most cases, the donor history questionnaire (DHQ) is completed before the tissue is procured; however, procurement could take place while DHQ is conducted as part of the DED. ^¶^At-risk processing takes place when TEs process tissue(s) pending the DED results used to make final determination regarding distribution. ^§^One or more tissue processors might be involved in the processing step depending on the procured tissue(s) and the requesting TEs. *DED* Donor eligibility determination, *DHQ* donor history questionnaire, *OPO* Organ Procurement Organization, *OPTN* Organ Procurement and Transplantation Network, *TC* transplant centre, *TEs* tissue establishments

Figures 1 and 2 illustrate the complex process of OTE donation and transplantation. This context is essential to understanding the challenges encountered in obtaining data and subsequently, TODES results. OTE donation processes are similar except for some differences in the timing of donor screening and testing, as organs and eyes must be recovered and transplanted in a short time window, whereas most tissues can be stored for longer periods of time.

## Results

Table 2 summarizes all records received by TODES, stratified by organization type (OPO or eye bank) and participant (A–M and E–J, where the letters are used to deidentify the organization). The TODES database contains 291 848 records received from nine OPOs and 42 451 records received from four eye banks. The majority of the donor records are males (average 63%, range 62.6–63.9%). Most of the OPO records received (88%, 255 927/291 848) do not indicate donor type; of those, 254 050 (87%) are from two participants (B and L). About 1% (487/42 451) of the records received from eye banks have no indication of donor type. The link between records received from OPO and United Network for Organ Sharing (UNOS) is indicated in Table 4. While 8141 OPO records can be linked to OPTN donor data received from UNOS, 1158 UNOS records with IDs in the OPTN data cannot be linked to OPO data [OPO absent (−), UNOS present (+); Table 4]. This mismatch occurs because OPOs typically assign a UNOS ID when a patient is identified as a potential donor, but many potential donors fail to become candidate organ donors during the screening process. However, some of those candidates may still become tissue donors, depending on the reason for not recovering organs. Records of actual organ donors are provided to UNOS; a total of 88% (range: 60.2–100%; OPO+, UNOS+; Table 5) of records in UNOS dataset were found in datasets shared by OPOs.

**Table 4:** Linkage between data from UNOS and OPOs

Organization code	Total records in UNOS dataset	Record count: OPO+, UNOS−	Record count: OPO−, UNOS+	Record count: OPO+, UNOS+ (%)	Record count: discrepant data in variable fields
A	1248	4860	497	751 (60.2)	229
B	1382	168 379	1	1381 (99.9)	29
C	654	3315	2	652 (99.7)	0
D	629	1249	11	618 (98.3)	57
F	1135	7167	0	1135 (100)	1
I	1458	2868	92	1366 (93.7)	490
K	644	116	0	644 (100)	50
L	724	74 534	0	724 (100)	0
M	1425	91	555	870 (61.1)	0

Source: adapted from TODES report

Data records provided by each OPO were merged with records obtained from UNOS. The linkage between data from UNOS and OPOs was produced from merging the data records

% means percent of all the records found in UNOS that were matched with OPO; +  signifies that it is present; − signifies that it is absent

*OPO* organ procurement organization, *UNOS* United Network for Organ Sharing

**Table 5:** Characteristics of potential donors in the TODES database by year

Year	Records *n*	Male (%)	Female (%)	Organ donors (%)^*^	Tissue donors (%)^†^	Eye donors (%)	Consent/authorization documented (%)
2009	12 871	62.6	37.4	10.2	51.3	74.0	97.2
2010	13 527	63.3	36.7	10.5	53.1	71.5	97.4
2011	14 198	63.9	36.1	13.3	54.1	71.7	94.1
2012	16 272	63.3	36.7	11.5	49.9	74.0	91.8
2013	17 876	63.2	36.8	11.1	49.7	75.1	90.1
2009–2013	74 744	63.3	36.7	11.3	51.5	73.4	93.8

Source: adapted from TODES report

Based on all records received; excludes known duplicates

^*^Percentage of donors with at least one organ recovered for transplant

^†^Percentage of donors with at least one tissue recovered for transplant

^¶^Percentage of donors with at least one ocular tissue recovered for transplant

About 8% (23 510/291 848; range 1–100%) of total OPO records received had at least one infectious disease test result compared with 93% (40 465/42 451; range 89–99%) of total eye bank records received (Table 2). The records with any test results were stratified into three groups by donor type (organ donors, tissue donors and eye donors); if donor type was not indicated, it was defined as a referrals-only record for any test results. Such low percentages of referrals-only records (for example, no organs were recovered) with at least one test in an OPO dataset occurred because tissue donor test results were not commonly shared with the OPO at that time, even when individuals from whom tissue was recovered were identified as tissue donors. If the individual was determined to be HIV, HBV, or HCV positive, the donated tissues were deemed ineligible for tissue transplantation purposes per FDA regulations.

Among the 23 510 records with test results in the OPO dataset, only 8149 (approximately 35%) were organ donor records; by contrast, among the 40 465 recorded in the eye-bank dataset, only 203 (< 1%) were organ donors. For tissue-donor records with at least one test result, 18 105 records were from OPOs and 11 665 from eye banks. For eye-donor records only, 6365 were from OPOs and 40 189 from eye banks. A small number of referrals-only records, 655 from OPOs and 276 from eye banks, had at least one test result. In this study, the referrals-only data were ultimately excluded from analysis because (a) most did not have any test results associated with the test data, and (b) the data came from a subset of the organizations in the dataset. This indicates that, with the current system, reliable data cannot be obtained about all potential organ or tissue donors, which is important information contributing to understanding the baseline infectious disease risks associated with OTE donor populations. As a result, data were analysed only from deceased donors with at least one recovered organ, tissue or ocular tissue with the intent to transplant.

The 2013 Public Health Service (PHS) guideline [14] provided 11 risk factors associated with an increased likelihood of recent HIV, HBV or HCV infection amongst organ donors. Organ donors with one or more of these risk factors were identified as at “increased risk” for infections in the UNOS dataset. Table 6 summarizes the increased-risk indicator stratified by year in the UNOS dataset, which was available for almost all organ donors (99.8%). According to the 5-year-period dataset, 10.1–16.2% of organ donors per year (with an average of 13.9%) were classified as increased-risk donors. Donors missing this information did not exceed 0.2% per year. Donating with a risk factor is permissible only for organ donors. Per 21 Code of Federal Regulations (CFR) Part 1271 (not applicable to organs), potential tissue (including ocular) donors with risk factors for certain infectious diseases are ineligible for donation, so data regarding “increased risk” donors for tissue and eye donation do not exist.

**Table 6:** Infectious disease risk status^*^ of organ donors by year

Year	Records, *n*^†^	Yes, *n* (%)	No, *n* (%)	Not done, *n* (%)	Missing, *n* (%)
2009	1266	128 (10.1)	1132 (89.4)	4 (0.3)	2 (0.2)
2010	1359	178 (13.1)	1179 (86.7)	0 (0.0)	2 (0.2)
2011	1830	250 (13.7)	1578 (86.2)	0 (0.0)	2 (0.1)
2012	1809	276 (15.3)	1532 (84.7)	0 (0.0)	1 (0.1)
2013	1877	304 (16.2)	1573 (83.8)	0 (0.0)	0 (0.0)
2009–2013	8141	1136 (13.9)	6994 (85.9)	4 (0.1)	7 (0.1)

Source: adapted from TODES report

Risk status of infectious disease was obtained from UNOS data

^*^Infectious disease risk status is an assessment of risk for blood-borne disease transmission per 2010 PHS guidelines, that is, the organ donors who met one or more criteria considered as behavioural or medical risk factors for recent HIV infection

^†^Represents donor records from OPOs that can be linked to donor records received from UNOS

## Discussion and conclusions

This effort highlights multiple lessons learned regarding knowledge gaps and challenges of using RWD in assessing the rate of communicable diseases in potential OTE donors (which was selected as a test case to understand the ability to collect donor-related data within the existing OTE workflow) that can, in turn, be used to better inform policy decision-making. Importantly, findings from the data captured by TODES led to precluding its use for policy decisions. First, the data were determined to not be fit-for-purpose, which was not surprising given the data provided by the organizations were collected largely to support business operations rather than to address research and surveillance questions. Specifically, available RWD data could not identify duplicate data among tissue donors, provide a tissue donation denominator or ascertain a representative sample of donors. Second, it was not possible to ascertain a comprehensive understanding of the true infectious disease status of actual donors, and what is particularly lacking are data on potential donors found to have communicable disease risk and therefore the donation did not proceed. From a testing perspective, supplemental (that is, “confirmatory”) tests were rarely performed to verify positive or indeterminate test results, presumably because of the lack of appropriately labelled supplemental tests, lack of adequate specimen volume, inability to sequentially follow deceased donors to document the evolution of infectious disease test markers and lack of regulatory or policy requirements to perform supplemental testing. Furthermore, testing data of potential non-transplantation donors – for example, that likely had positive test results or identified communicable disease risks – were largely unavailable, and if available, data were incomplete. Third, the various testing protocols that estimated infectious disease marker prevalence lacked standardization and included a variety of assay types such as donor screening and diagnostic assays (ST 1). Fourth, donors from OPO- and TE-evaluated datasets cannot be uniquely identified in large part because donors lack a common identifier between the organ and tissue/eye transplantation pathways as well as within the tissue/eye pathway when tissues go to more than one establishment. As such, any assembled dataset contains a mixture of test results (that is, positive results with no further testing, inconclusive results with no further testing, positive results with subsequent testing and negative results with subsequent testing), severely impacting the interpretability and usefulness of the data.

TODES focused on available OTE donor data that might be used to characterize the donor populations to then inform donor screening and testing policy. However, acknowledging how these data are part of biovigilance within an interconnected system needed to maximize overall OTE transplantation safety is also important. Multiple facets contribute to the biovigilance required to maximize transplantation safety [15]. These include (i) donor selection (defining and identifying potential donors, donor screening and testing information), (ii) careful manufacturing practices (processing practices that both prevent contamination and cross-contamination and that remove or inactivate contamination to the extent possible while maintaining utility of the product) and (iii) identifying and investigating adverse events (to learn about the causes to inform improved policies and practice, and to quickly identify other recipients to prevent further adverse event occurrence). These facets of transplant safety require traceability of tissues from the time of considering a potential donor all the way through to the transplantation/implantation to a recipient. Ideally, donor evaluation data and transplantation outcomes data collection could also be used as part of efforts to proactively identify emerging infectious disease threats. The TODES study participants agreed that interventions that can yield benefits to transplantation safety include better communication, better identification methods, better education, etc. A comprehensive list is presented in ST 2. These interventions are consistent with the conclusions of the Transplantation Transmission Sentinel Network (TTSN) pilot program that was developed to collect data on donation, tissue implantation and adverse events [16]. The authors of the TTSN program concluded that eye and tissue tracking from recovery to implantation will be necessary before a sentinel network system can be operable, which would require common identifiers and nomenclature. They further stated that the absence of a US sentinel network may result in future transmission events that could have been otherwise preventable. There is a clear need for such an integrated system for OTE transplantation data collection.

### Study limitations

TODES had some limitations. The study was designed to solely address the ability to collect RWD in characterizing the baseline communicable disease epidemiology of potential OTE donors, and was not designed to address any identified challenges, explore other sources of risk from tissue manufacturing, evaluate communicable disease transmission data, or to make policy recommendations. Data integration from different sources (for example, eye banks, large TEs and large laboratories that provide infectious disease testing) was challenging, which resulted in excluding those sources and acquiring tissue donor data only from OPOs that were also tissue recovery establishments. Thus, TODES data are not representative of national organ and tissue donor/donation data. Also, the 2009–2013 donor data collected by TODES reflect the recommendations in the 1994 PHS guidelines [17] that do not address later guideline development (Table 1) in defining “increased risk” organ donors. While the 1994 PHS guidelines were designed to reduce the risk of HIV transmission by screening organ and tissue donors to capture behaviours and medical history placing them at increased risk for HIV infection [17], the subsequent 2013 PHS guideline, limited to organ donors [14], recommends additional donor and recipient screening for HBV and HCV, including use of more sensitive testing methodologies, revised risk factors and more robust informed consent discussions about accepting or rejecting organs from donors known to be infected with HBV or HCV. A new PHS guideline published in June 2020 [18], and was implemented by 1 March 2021 [19, 20]. Changes to the PHS guideline over time highlight two important issues: (1) the same donor data are reviewed when evaluating organ and tissue donors, but differing risk benefit ratios may rightfully result in different donor decisions between organs and tissues/eyes, and (2) it is important to carefully consider unintended availability consequences as the result of any policy or guidance changes [21].

### Future needs

TODES highlighted the need for a more integrated system for accessing and collecting all OTE donor evaluation data (that is, including data for all potential donors, not just data on donors who were found eligible to donate). Although more burdensome data entry requirements could be considered in policy and regulations, such data entry is currently manual, time consuming and subject to transcription error. The Retrovirus Epidemiological Donor Studies (REDS) research program, which aims to evaluate and propose models for improving the safety of blood donations, provides a model that addresses key challenges in organ and tissue safety. Over the course of 30 years, the National Heart, Lung, and Blood Institute (NHLBI) established the Retrovirus Epidemiological Donor Studies (REDS-I, 1989–2001 [22], REDS-II, 2004–2012 [23]) and the subsequent Recipient Epidemiology and Donor Evaluation Studies (REDS III 2011–2018 [24] and REDS-IV-P 2019–2026 [25]) to conduct research on infectious disease risks to the safety and availability of the blood supply, and with REDS-III and -IV-P, linking donated blood to the safety and effectiveness of transfusions. At the time of the funding of REDS-1 (1989), questions arose about the residual risk of infectious diseases, including HIV, HCV and HBV in the blood supply. Using a distributed research model, multiple entities (blood collectors, hospitals, testing centres and analytical coordinating centres) contributed and analysed data and biospecimens to track blood safety. As a result of the REDS program, donor testing platforms have matured, and new threats have been identified and investigated (for example, West Nile Virus).

Over this time, the risks of acquiring HIV or HCV infection through transfusion have decreased from about 1:200 000–300 000 donations to 1:1.5–2.0 million donations [10]. Much of the decline was attributed to nucleic acid testing (NAT), which was implemented on the basis of data from REDS protocols and analyses of comprehensive donor and donation data captured from participating blood centres. Consequently, the REDS studies have informed regulatory decision-making and public health policies for more than a quarter century. This type of research program enables quick assessment of blood safety risks after a new threat or pathogen has emerged.

Such a model could be used to establish a baseline of infectious risk among OTE donor populations, enabling the evaluation of risk/benefit of interventions upon identification of new threats. To build such an integrated transplant data collection system, an appropriate funding sponsor should be identified, harmonized definitions and testing approaches should be established, unique donor identifiers should be assigned, labelling to facilitate traceability should be implemented, and OPO, TE and eye bank engagement should be assured. In addition, hospitals and clinician users of tissues and organs must understand the need to populate the system with additional data (that is, improve recording of tissue provided to patients, respond to information requests and tissue utilization cards provided by TEs and monitor for and promptly report potential recipient adverse events) [7], the value of additional research and the associated costs. There is no standard for data collection; different establishments use their own systems for data collection. Costs of establishing an integrated transplant data collection system can be daunting. However, standardized and interoperable data that would be used to streamline and optimize donor evaluation, prevent transmission events and identify transmission events quickly to facilitate rapid response to minimize recipient morbidity and mortality could likely decrease overall costs to TEs and the entire healthcare system over time.

Currently, these types of prospective data collection and analyses are viable, and as described below, need not be unduly burdensome because of the evolution of healthcare IT. Automated data collection capacity has now far exceeded the data and testing infrastructure of the 1990s, 2000s and even 2010s. A system could be designed to prospectively collect the data required to estimate incidence, prevalence and risk factors of organ and tissue/eye donors. It could also provide input to benefit–risk assessment models supporting policy evaluation. As described above, data collection and analysis cannot be supported by the electronic information currently available and stored by the organizations surveyed. As described and consistent with the TTSN experience, these issues reinforce the need to involve all stakeholders in the standards and systems development process to ensure the availability and accuracy of the appropriate and consistently defined data elements.

### Healthcare IT solutions

Recent healthcare information technology (IT) advancements [26, 27] position RWD as a potential prospect for better informing policy and regulatory decision-making, even as the current system for collecting and tracking donor data remains largely unchanged. The intersection of the following three factors gives rise to RWD as a potential solution: (1) increasing adoption of electronic health records (EHRs) [28]; (2) emerging HL7^®^ Fast Healthcare Interoperability Resources (FHIR^®^) standards [29]; and (3) 21st Century Cures Act mandates to promote interoperability with FHIR R4 as the standard [30]. Embedding information about both the donation/recovery and the transplantation with specific biologically derived product (BDP) codes and donor identifiers in the EHR would enable forward and backward traceability from an impacted (for example, infected) patient or product of concern to other patients or products from the same donor. The US Core Data for Interoperability, the standardized set of health data classes and data elements, is poised to include BDP in a future release, requiring all EHR systems to make these data available to other systems, including those internal and external to the transplanting hospital provider.

The changing landscape of healthcare IT and the continuous development of interoperability standards are the foundation for a sustainable and robust solution to improve organ and tissue safety. Therefore, it is paramount to invite all stakeholders to discuss how these data can be streamlined by standardizing, capturing, storing and transmitting quickly and confidentially to establish RWD for the purposes of donor-to-recipient traceability and to improve transplantation safety.

## Supplementary Information

Additional file 1.

## Data Availability

All data collected and analysed for TODES are summarized in the publicly available report at https://www.hhs.gov/sites/default/files/tissue-and-organ-donor-epidemiology-study.pdf.

## Abbreviations

BDP: Biologically derived product
CDC: Centers for Disease Control and Prevention
DED: Donor eligibility determination
DHQ: Donor history questionnaire
FDA: Food and Drug Administration
EHR: Electronic health record
HHS: Health and human services
HRSA: Health Resources and Services Administration
TODES: Tissue and Organ Donor Epidemiology Study
HIV: Human immunodeficiency virus
HBV: Hepatitis B virus
HCV: Hepatitis C virus
NAT: Nucleic acid amplification test
OPO: Organ procurement organization
OPTN: Organ procurement and transplantation network
REDS: Retrovirus Epidemiological Donor Studies
RWD: Real-world data
TC: Transplant centre
TE: Tissue establishment
TTSN: Transplantation Transmission Sentinel Network
UNOS: United Network for Organ Sharing
WG: Working group

## Footnotes

1 The Center for Biologics Evaluation and Research (CBER) regulates the human cells, tissues and cellular and tissue-based products (HCT/Ps) under 21 Code of Federal Regulations (CFR) Parts 1270 and 1271.
