# Supplementary material for: Real-world data to improve organ and tissue donation policies: lessons learned from the tissue and organ donor epidemiology study
Source: Health Res Policy Syst. 2024 Nov 12;22:152. doi: 10.1186/s12961-024-01237-0 (PMC11556174; doi:10.1186/s12961-024-01237-0)
Supplement: Supplementary file 1 — Additional file 1. [file 12961_2024_1237_MOESM1_ESM.docx]

**Supplementary Material**

ST 1 Donor Eligibility Screening Practices by organization

| OPOs | 5 OPOs utilized the services of Statline® to provide all initial screening for donor eligibility  3 OPOs used Statline® for back- up screening  11 OPOs conducted donor screening in-house  (one of the 11 OPOs also utilized answering service through another OPO, as needed) |
| --- | --- |
| Tissue Banks | ~95% (19/20) of OPOs provided donor screening and recovery services to TEs.  On average each OPO serves 4 banks (range: 1-6 banks)  About 5% of tissue banks conduct their own donor screening, while OPOs recover the tissues. |
| Eye Banks | ~90% of OPOs provided donor screening services for eye banks  ~50% of OPOs provided recovery services. |

Source: TODES report

Lack of standardization in the organizations testing protocols to estimate infectious disease prevalence.

Abbreviations: OPO, organ procurement organization; TE, tissue establishmen

ST 2 Summary of important interventions

| **Proposed Interventions** |
| --- |
| Communication networks should be improved |
| A unique donor identifier for both organs and tissues should be created |
| Education and dissemination of information to clinicians and transplant patients should be strengthened. |
| A framework for clinicians to report transplant-associated adverse events should be clearly delineated |
| A notification algorithm for tracking among and between organs and tissues should be designed |

Source: TODES report

The summary is based on identified challenges ([1](#_ENREF_1), [5](#_ENREF_5), [42](#_ENREF_42)). From the preliminary solutions and concepts suggested, the TODES participants agreed upon the above as the most important interventions that can yield benefits on a relatively short-term basis.
